# Preoperative prealbumin-to-fibrinogen ratio to predict survival outcomes in hepatocellular carcinoma patients after hepatic resection

**DOI:** 10.5937/jomb0-32980

**Published:** 2022-07-29

**Authors:** Haixi Yan, Shuaishuai Chen, Yang Qiong, Linling Cai

**Affiliations:** 1 Zhejiang Chinese Medical University, Hangzhou, Zhejiang Province, China; 2 Taizhou Hospital of Zhejiang Province affiliated to Wenzhou Medical University, Department of Clinical Laboratory, Linhai, Zhejiang Province, China

**Keywords:** hepatocellular carcinoma, prealbumin-to-fibrinogen ratio, survival analysis, nomogram, hepatocelularni karcinom, odnos prealbumina i fibrinogena, analiza preživljavanja, nomogram

## Abstract

**Background:**

This study aimed to evaluate the clinical application of the preoperative prealbumin-to-fibrinogen ratio (PFR) in the clinical diagnosis of hepatocellular carcinoma (HCC) patients and its prognostic value.

**Methods:**

The clinical and laboratory data of 269 HCC patients undergoing surgical treatment from January 2012 to January 2017 in Taizhou Hospital were retrospectively analysed. The Cox regression model was used to analyse the correlation between the PFR and other clinicopathological factors in overall survival (OS) and disease-free survival (DFS).

**Results:**

Cox regression analysis showed that the PFR (hazard ratio (HR)=2.123; 95% confidence interval (95% CI), 1.271-3.547; P=0.004) was an independent risk factor affecting the OS of HCC patients. Furthermore, a nomogram was built based on these risk factors. The C-index for the OS nomogram was 0.715.

**Conclusions:**

Nomograms based on the PFR can be recommended as the correct and actual model to evaluate the prognosis of patients with HCC.

## Abstract (sr)

**Uvod:**

Cilj ove studije je bio da proceni kliničku primenu preoperativnog odnosa prealbumin-fibrinogena (PFR) u kliničkoj dijagnozi pacijenata sa hepatocelularnim karcinomom (HCC) i njegovu prognostičku vrednost.

**Metode:**

Sprovedena je retrospektivna analiza kliničkih i laboratorijskih podataka 269 pacijenata sa HCC koji su bili na hirurškom lečenju od januara 2012. do januara 2017. u Taizhou bolnici. Za analizu korelacije između PFR-a i drugih kliničko-patoloških faktora u ukupnom preživljavanju (OS) i preživljavanju bez bolesti (DFS) korišćen je Koksov regresioni model.

**Rezultati:**

Koksova regresiona analiza je pokazala da je PFR (indeks rizika (HR) = 2,123; 95% interval pouzdanosti (95% CI), 1,271-3,547; P = 0,004) bio nezavisni faktor rizika koji je uticao na ukupno preživljavanje pacijenata sa HCC. Dodatno, na osnovu ovih faktora rizika je urađen nomogram. C-indeks za nomogram ukupnog preživljavanja je bio 0,715.

**Zaključak:**

Nomogrami zasnovani na PFR-u se mogu preporučiti kao ispravan i stvarni model za procenu prognoze kod pacijenata sa HCC.

## Introduction

Hepatocellular carcinoma (HCC) is a globally prevalent malignant tumour with a 5-year survival rate (<5%) [1]. HCC is mainly diagnosed by imaging examinations, serological markers (alpha-fetoprotein, a-L-fucosidase, abnormal prothrombin, and so on), and liver histopathological diagnoses. Moreover, China has the highest incidence of liver cancer. According to informal statistics, the number of Chinese people who die of liver cancer each year is approximately 110,000, accounting for 45% of the world's liver cancer deaths [2].

Fibrinogen is an important acute-phase protein. Recent studies have found that fibrinogenaemia is strongly related to various tumours' occurrence, development, and prognosis. These tumours mainly include renal cell carcinoma, lung cancer, ovarian cancer, and hepatocellular carcinoma [3] [4] [5] [6]. Furthermore, prealbumin levels can often be used to assess a patient's nutritional status. Consequently, prealbumin levels can reflect postoperative effects such as cervical cancer, metastatic renal cell carcinoma, and non-small-cell lung cancer [7] [8] [9]. Furthermore, a new prognostic indicator has been reported, which is a combination of prealbumin and fibrinogen. Several articles have shown that prealbumin and fibrinogen (PFR) ratio has good prognostic significance in acute pancreatitis [10].

Therefore, the trend of early detection and early intervention is crucial for HCC treatment and mortality reduction. Finding new HCC biomarkers to predict the prognosis of HCC patients after surgery is urgent. Thus, this study aimed to use nomograms to analyse the relationship of the preoperative PFR in the prognosis of HCC patients to determine its prognostic value in HCC.

## Materials and methods

### Patients

This study recruited 367 HCC patients admitted to the Department of Hepatobiliary Surgery of Taizhou Hospital of Zhejiang Province, Taizhou, China, from January 2012 to January 2017. The inclusion criteria were as follows: (a) complete clinical and laboratory data (current medical history, previous medical history, family history, personal history, physical examination, and complete pathological data); (b) abdominal computed tomography or ultrasound to exclude liver abscess, liver-occupying lesions, and other diseases; (c) the use of required pathological data to exclude secondary liver cancer; (d) postoperative TNM clinical-pathological stages I, II, and III; and (e) exhaustive whole blood cell analysis, biochemical analysis, and examinations. The preoperative clinical diagnosis was clear, and the tumour location, size, number, pathological stage, and differentiation degree were obtained through pathological examination. The intraoperative lymph nodes were examined to determine the lymph node metastasis in each study group. Preoperative blood examination data was exhaustive. Furthermore, the exclusion criteria were (a) patients with other benign liver tumours (e.g., haemangiomas and hepatic adenomas) and (b) patients with infections or other inflammatory diseases. Finally, 269 patients were included.

### Treatment and follow-up

All patients underwent a 1-year regular followup visit. The follow-up deadline was April 22, 2017. The recurrence and survival times were all recorded in months, from surgery to recurrence or death. According to the follow-up data criteria, tumour recurrence or metastasis was the DFS endpoint. Furthermore, the overall survival (OS) endpoint was death.

### Statistical analysis

The Statistical Package for the Social Sciences, version 22.0, and X-tile, version 3.6.1, were used for analysis. The optimal PFR cut-off value was calculated using the X-tile plot. The X″ test was used to analyse the differences between the categorical variables. Moreover, the Cox proportional hazards model was used for univariate and multivariate survival analyses to determine the risk factors for prognosis. Finally, the nomogram for prediction value was established using R software. Consequently, model accuracy was evaluated by Harrell's concordance index (c-index), calibration plots, decision curves, and clinical impact curves. *P* values ≤ 0.05 were considered statistically significant.

## Results

### Analysis and calculation of the PFR optimal cutoff value

**Figure 1:**
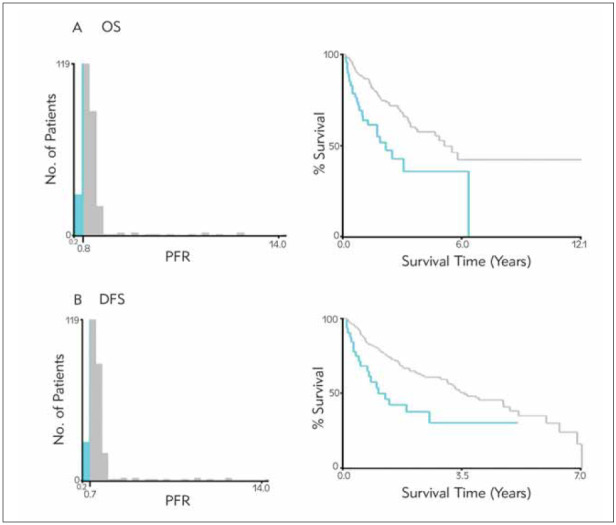
X-tile OS analyses were performed using patient data to determine the optimal PFR cut-off values. The optimal cutoff values are shown in the histograms of the entire cohort (left panels), and Kaplan–Meier plots are displayed (right panels). The P values were determined by using the cut-off values defined in the training sets and applying them to the validation sets. A. For OS, the optimal cut-off value of the PFR was 0.8. B. For DFS, the optimal cut-off value of the PFR was 0.7.

This study group enrolled 269 patients. Taking postoperative mortality of the patients as the endpoint, 0.8 was the optimal PFR cut-off value calculated by the X-tile plot (Figure 1).

### Patient characteristics

The patients' baseline and clinicopathological characteristics stratified by the PFR are described in Table 1. Furthermore, the table shows that the patients were divided into two groups for further analysis (PFR<0.8 and PFR 0.8). Moreover, the PFR was associated with the Barcelona Clinic Liver Cancer (BCLC) stage, Child-Pugh score, complications (hepatic rupture, portal hypertension, and intraoperative ascites), survival, tumour length, and red cell distribution width. The average age of the 269 patients was 57 years, with 219 (81.4%) males and 50 (18.6%) females. Moreover, patients with stages A and B-C, using the BCLC staging system, accounted for 55.8% and 44.2% of the total patients, respectively. However, patients with grades A and B accounted for 89.6% and 10.4% of the total patients' Child-Pugh scores, respectively. Patients with complications of liver rupture bleeding, portal hypertension, and hepatic encephalopathy accounted for 7.8%, 6.3%, and 0.7% of the total patients, respectively. Consequently, 173 patients survived (64.3%). Of the total patients, 167 (62.1%) and 102 (37.9%) were diagnosed with tumours <5 and 5 cm, respectively.

**Table 1:** Comparison of baseline clinicopathological characteristics based on PFR.

		Cases	PFR		*P*
		No. (%)	<0.8	≥0.8	
Age (years)	≤60	164 (61%)	25	139	0.312
	>60	105 (39%)	21	84	
Gender	Male	219 (81.4%)	35	184	0.308
	Female	50 (18.6%)	11	39	
Smoking	No	140 (52%)	25	115	0.731
	Yes	129 (48%)	21	108	
Drinking	No	196 (72.9%)	37	159	0.205
	Yes	73 (27.1%)	9	64	
BCLC stage	A	150 (55.8%)	18	132	0.013
	B, C	119 (44.2%)	28	91	
Child-Pugh score	A	241(89.6%)	31	210	0.000
	B	28 (10.4%)	15	13	
Liver rupture<br>bleeding	No	248 (92.2%)	33	215	0.000
	Yes	21 (7.8%)	13	8	
Portal hypertension	No	252 (93.7%)	37	215	0.000
	Yes	17 (6.3%)	9	8	
Hepatic encephalopathy	No	267 (99.3%)	45	222	0.215
	Yes	2 (0.7%)	1	1	
Recrudescence	No	146 (54.3%)	21	125	0.197
	Yes	123 (45.7%)	25	98	
Survival situation	Survival	173 (64.3%)	23	150	0.026
	Mortality	96 (35.7%)	23	73	
Cirrhosis	No	69 (25.4%)	7	62	0.075
	Yes	200 (74.3%)	39	161	
Liver capsule<br>invasion	No	220 (81.8%)	37	183	0.795
	Yes	49 (18.2%)	9	40	
Liver margin	No	247 (91.8%)	39	208	0.056
	Yes	22 (8.2%)	7	15	
Nerve invasion	No	259 (96.3%)	45	214	0.543
	Yes	10 (3.7%)	1	9	
Vascular invasion	No	201 (74.7%)	30	171	0.103
	Yes	68 (25.3%)	16	52	
T stage	T0–T1	157 (58.4%)	24	133	0.350
	T2–T4	112 (41.6%)	22	90	
N stage	N0	264 (98.1%)	45	219	0.862
	N1	5 (1.9%)	1	4	
M stage	M0	263 (97.8%)	45	218	0.977
	M1	6 (2.2%)	1	5	
Tumour size (cm)	<5 cm	167 (62.1%)	20	147	0.004
	5 cm	102 (37.9%)	26	76	
Number of<br>tumours	1	209 (77.7%)	38	171	0.379
	2	60 (22.3%)	8	52	
Hepatitis B<br>infection	No	47 (17.5%)	43	4	0.097
	Yes	222 (82.5%)	181	41	
PLT	300 × 10^9^/L	259 (96.3%)	45	214	0.543
	>300 × 10^9^/L	10 (3.7%)	1	9	
AFP	20 mg/L	116 (43.1%)	15	101	0.114
	>20 mg/L	153 (56.9)	31	122	
CEA	≤5 ng/mL	232 (86.2%)	41	191	0.533
	>5 ng/mL	37 (13.8%)	5	32	

### Prognostic value of the PFR

Univariate analysis was carried out using the Cox regression model. Consequently, the clinicopathological parameters predicting OS and DFS were further studied. In the univariate analysis, the BCLC stage, nerve invasion, vascular invasion, T stage, M stage, tumour size, number of tumours and PFR were significantly related to OS (*p*<0.05). Furthermore, the BCLC stage, portal hypertension, vascular invasion, T stage, M stage, tumour size, number of tumours and PFR were associated with DFS (*p*<0.05). For the multivariate Cox regression OS model, the PFR (hazard ratio (HR)=2.123; 95% confidence interval (95% CI), 1.271-3.547; *P*=0.004), vascular invasion (HR=2.272; 95% CI, 1.032-5.003; *P*=0.041), M stage (HR=8.095; 95% CI, 3.518-18.627; *P*=0.000), and tumour size (HR=2.188; 95% CI, 1.240-3.859; *P*=0.007) were verified to be independent prognostic factors in patients with HCC (Table 2).

**Table 2:** Univariate and multivariate survival analyses of OS and DFS in HCC patients.

	OS				DFS			
	Univariate analysis<br>HR (95% CI)	*P*	Multivariate analysis<br>HR (95% CI)	*P*	Univariate analysis<br>HR (95% CI)	*P*	Multivariate<br>HR (95% CI) analysis	*P*
Age (years)		0.489		0.389		0.937		0.525
≤60	1.000		1.000		1.000		1.000	
>60	1.157<br> (0.766–1.749)		1.010		0.985<br> (0.680–1.427)		1.133<br> (0.772–1.663)	
Gender		0.948		0.796		0.985		0.991
Male	1.000		1.000		1.000		1.000	
Female	0.982<br> (0.574–1.682)		0.927<br> (0.520–1.651)		0.995 <br>(0.621–1.595)		0.997<br> (0.614–1.621)	
Smoking		0.545				0.424		
No	1.000				1.000			
Yes	1.132 <br>(0.758–1.690)				1.157<br> (0.809–1.653)			
Drinking		0.536				0.386		
No	1.000				1.000			
Yes	1.146<br> (0.744–1.766)				1.189 <br>(0.804–1.757)			
BCLC stage		0.000		0.421		0.001		0.852
A	1.000		1.000		1.000		1.000	
B, C	2.171 <br>(1.445–3.263)		0.766 <br>(0.401–1.466)		1.800<br> (1.257–2.579)		0.945<br> (0.523–1.709)	
Child’s score		0.055				0.123		
A	1.000				1.000			
B	1.742<br> (0.988–3.073)				1.532<br> (0.891–2.635)			
Liver rupture bleeding		0.083				0.238		
No	1.000				1.000			
Yes	1.746 <br>(0.930–3.278)				1.433<br> (0.789–2.604)			
Portal hypertension		0.068				0.002		0.062
No	1.000				1.000		1.000	
Yes	1.842 <br>(0.955–3.551)				2.462 <br>(1.378–4.397)		1.870 <br>(0.970–3.604)	
Hepatic encephalopa-		0.547				0.727		
No	1.000				1.000			
Yes	1.833 <br>(0.255–13.188)				1.421 <br>(0.198–10.193)			
Cirrhosis		0.699				0.403		
No	1.000				1.000			
Yes	1.100 <br>(0.678–1.787)				1.203 <br>(0.780–1.858)			
Liver capsule invasion		0.328				0.881		
No	1.000				1.000			
Yes	1.270<br> (0.787–2.048)				1.035<br> (0.661–1.621)			
Liver margin		0.085				0.234		
No	1.000				1.000			
Yes	1.782<br> (0.923–3.439)				1.459 <br>(0.784–2.717)			
Nerve invasion		0.034		0.330		0.168		
No	1.000		1.000		1.000			
Yes	2.456 <br>(1.071–5.633)		1.596 <br>(0.623–4.086)		1.787 <br>(0.783–4.078)			
Vascular invasion		0.000		0.041		0.001		0.151
No	1.000		1.000		1.000		1.000	
Yes	2.472<br> (1.619–3.774)		2.272<br> (1.032–5.003)		1.938<br> (1.315–2.856)		1.709<br> (0.823–3.550)	
T stage		0.000		0.938		0.001		0.778
T1	1.000		1.000		1.000		1.000	
T2, T3, T4	2.111<br> (1.412–3.157)		0.966<br> (0.408–2.286)		1.823 <br>(1.276–2.603)		0.894 <br>(0.411–1.947)	
N stage		0.876				0.623		
N0	1.000				1.000			
N1	1.119<br> (0.272–4.598)				0.698<br> (0.167–2.925)			
M stage		0.000		0.000		0.000		0.000
M0	1.000		1.000		1.000		1.000	
M1	13.114 <br>(5.515–31.184)		8.095<br> (3.518–18.627)		8.556 <br>(3.679–19.897)		5.015 <br>(2.253–11.163)	
Tumour length (cm)		0.000		0.007		0.001		0.073
<5 cm	1.000		1.000		1.000		1.000	
≥5 cm	2.320 <br>(1.551–3.471)		2.188 <br>(1.240–3.859)		1.868 <br>(1.306–2.671)		1.611<br> (0.957–2.711)	
Number of tumours		0.025		0.224		0.004		0.103
1	1.000		1.000		1.000		1.000	
≥2	1.658<br> (1.067–2.577)		1.532 <br>(0.770–3.048)		1.770 <br>(1.197–2.617)		1.714 <br>(0.896–3.278)	
Hepatitis B infection		0.630				0.674		
No	1.000				1.000			
Yes	0.816<br> (0.357–1.867)				0.849 <br>(0.395–1.822)			
PFR		0.001		0.004		0.007		0.092
≥0.8	1.000		1.000		1.000		1.000	
<0.8	2.261 <br>(1.411–3.624)		2.123<br> (1.271–3.547)		1.829 <br>(1.175–2.848)		1.525<br> (0.934–2.491)	

### Survival PFR analysis

**Figure 2:**
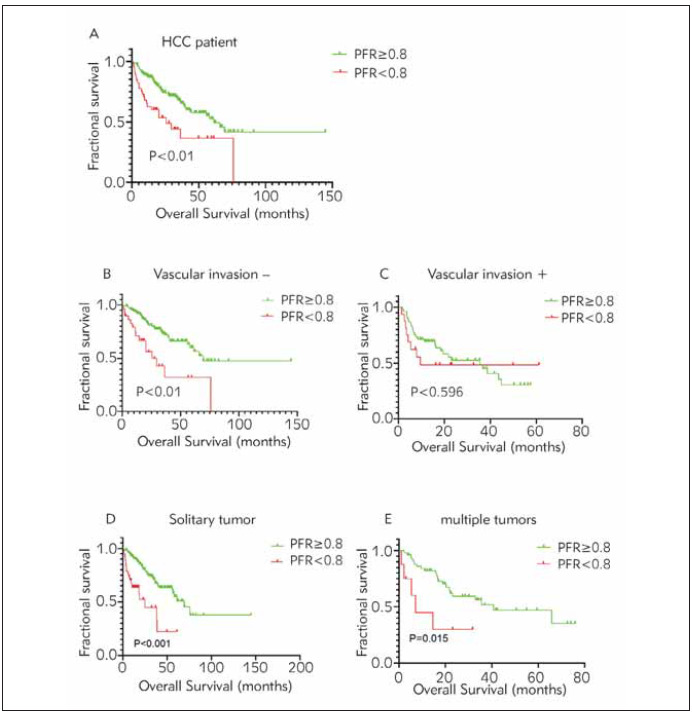
Kaplan–Meier curves for OS according to the PFR in each subgroup and the total HCC patients. A. total HCC patients, B. vascular invasion-negative subgroup, C. vascular invasion-positive subgroup, D. solitary tumour subgroup, and E. multiple tumours subgroup.

This study used Kaplan-Meier analysis to determine the prognostic value of the PFR. Low PFR levels (<0.8) were associated with short OS (Figure 2A). Based on vascular invasion and the number of tumours, a separate subgroup analysis was also conducted to investigate the significance of the PFR for the prognosis of HCC patients. Moreover, short OS was found in patients with low PFR in the solitary and multiple tumour subgroups (*P*<0.001 and *p*<0.001, respectively) and the vascular invasion-negative subgroup (*P*<0.001) but not in the vascular invasionpositive subgroup (*p*=0.596; Figure 2B, Figure 2C, Figure 2D, Figure 2E).

### Development and validation of nomograms for predicting OS in HCC patients

**Figure 3:**
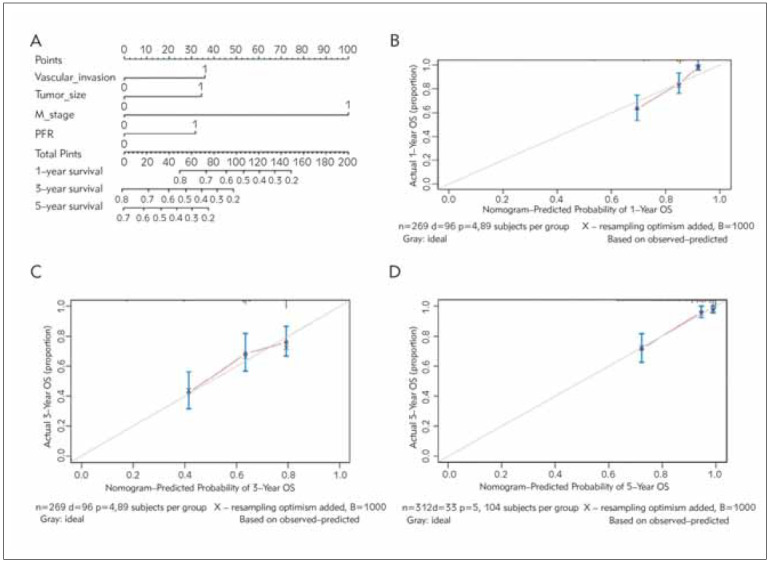
The PFR was an independent predictive factor for disease progression in HCC patients. A. Nomogram predicting the OS of HCC patients. B. 1-year OS calibration plot. C. 3-year OS calibration plot. D.5-year OS calibration plot.

**Figure 4:**
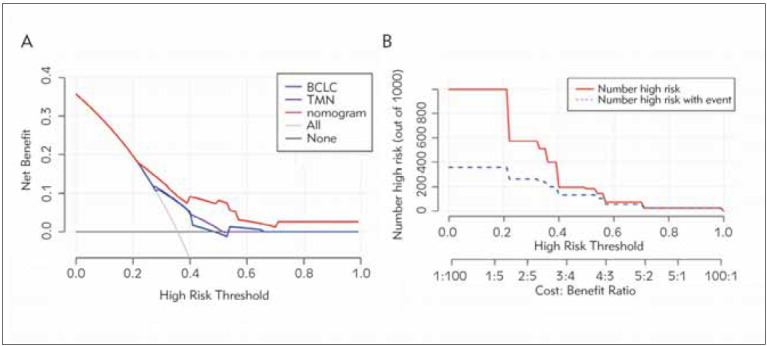
Decision curve for the disease progression of HCC patients. A. Nomogram, red line; BCLC stage, blue line. TMN stage, purple line. The abscissa of this graph is the threshold probability, and the ordinates are the net benefit. B. Clinical Impact Curve.

The nomograms can be explained by adding up the number of points assigned to each variable at the top of the scale. At the bottom of the scale, the total score translates into predicting the patient's 5-year probability of mortality. A nomogram, based on independent risk factors (the PFR, vascular invasion, M stage, tumour size), was established to predict OS in HCC patients (Figure 3A). The C-index for the OS nomogram was 0.715 (95% CI, 0.662-0.768). Moreover, the calibration curves by internal validation demonstrated good agreement between the predicted and actual probability of 1-, 3- and 5-year OS (Figure 3B, Figure 3C, Figure 3D). The decision curve analysis found that the nomogram model that included the PFR had better net benefits than the model that included the BCLC and TNM stages to identify OS for HCC patients (Figure 4).

## Discussion

Malnutrition and fibrinogen abnormalities are common in cancer patients and have major effects on their quality of life, treatment outcomes, and prognosis [11] [12]. This study indicates that a low preoperative PFR (<0.8) is an independent risk factor for OS in HCC patients.

Chronic infections, including HCC, cause >15% of malignancies worldwide [13]. Studies have shown that systemic inflammatory responses boost angiogenesis and tumour invasion by upregulating cytokines [14] [15] [16]. This shows that the inflammatory response plays a critical role in tumorigenesis and tumour development. Furthermore, the two functions of prealbumin may be related to the occurrence and prognosis of tumours. The first is that inflammation is associated with decreased prealbumin levels in several studies [17] [18]. Moreover, prealbumin levels may be affected in other ways during inflammation because cytokines (e.g., IL-6, IL-1, and TNF-a) can downregulate synthesis [19] and increase vascular permeability [20]. The second is that prealbumin can respond to the nutritional status of the reaction body. Serum prealbumin has a shorter half-life than albumin and is synthesised by hepatocytes. However, synthesis rapidly declines when hepatocytes are damaged. Furthermore, tumours cause protein malabsorption in the body and can also cause prealbumin levels to decline. Studies have recently shown that hypoproteinaemia is a poor prognostic indicator of oesophageal and colorectal cancers [21] [22].

Fibrinogen is the most acutely reactive plasma protein [1]. It plays an important role in activating the coagulation cascade [23]. Moreover, fibrinogen is also the key factor in regulating the inflammatory cascade through the interaction of ligand-receptor mechanisms involving immune cells (e.g., monocytes and microvasculature) [2] [24] [25]. Some studies have indicated that fibrinogen can be endogenously synthesised by cancer cells [26] [27]. Meanwhile, highly concentrated fibrinogen induces epithelial-mesenchymal transition, which increases cancer cell invasion and metastasis using a cell line model by increasing vimentin expression and decreasing E-cadherin expression [28].

Therefore, in theory, prealbumin and fibrinogen are two valuable markers for monitoring HCC progression. Furthermore, this study showed that the PFR, an inflammatory marker, is a potential prognostic factor for HCC patients, combining the two factors of HCC patients' nutritional status and inflammatory response status and having the advantages of low cost and convenience, rapidity, and easy detection.

This study tried to explain the prognostic value of the PFR for HCC patients. X-tile was used to calculate the optimal PFR cut-off value of 0.8. Moreover, many studies previously used the receiver operating characteristic (ROC) curve to select the cut-off value. However, most of the ROC curves included only the event outcomes and experimental indicators but did not include important factors for cancer prognosis. The X-tile software precisely includes the time worth choosing as the cut-off value. Thus, the cut-off value of this article may be more accurate. Furthermore, this study suggests that the PFR is associated with OS and DFS in HCC patients in univariate analysis, and the PFR is related to the patient's OS (HR=2.123; 95% CI, 1.271-3.547; *P*=0.004) and has nothing to do with DFS in multivariate analysis. The risk of mortality of HCC patients with a low PFR is significantly increased. Moreover, the nomogram was used to establish the prognostic model of liver cancer, and the accuracy of the nomogram was proven by calibration, decision, and clinical influence curves. The nomogram shows that the PFR has an important predictive value.

Although the PFR can predict OS in HCC patients, there are some limitations in this study. First, this single-centre retrospective study may have selection bias as it only included HCC patients undergoing surgical resection. Moreover, this study does not represent HCC patients who refuse surgery for different reasons. Second, verification queues are lacking to verify whether the findings of this study are commonly used. Therefore, the results of this study need to be further verified in forward-looking and large-scale cooperative research.

## Conclusion

In conclusion, the results of this study suggest that the PFR (<0.8) is a prognostic indicator of OS in HCC patients. Thus, a PFR-containing nomogram can be used as a more practical model for evaluating OS in HCC patients.

## Dodatak

### Conflict of interest statement

All the authors declare that they have no conflict of interest in this work.
